# Direct Heme Transfer Reactions in the Group A *Streptococcus* Heme Acquisition Pathway

**DOI:** 10.1371/journal.pone.0037556

**Published:** 2012-05-23

**Authors:** Chunmei Lu, Gang Xie, Mengyao Liu, Hui Zhu, Benfang Lei

**Affiliations:** 1 Department of Physiology, Harbin Medical University, Harbin, People's Republic of China; 2 Department of Immunology and Infectious Diseases, Montana State University, Bozeman, Montana, United States of America; University of Edinburgh, United Kingdom

## Abstract

The heme acquisition machinery in Group A *Streptococcus* (GAS) consists of the surface proteins Shr and Shp and ATP-binding cassette transporter HtsABC. Shp cannot directly acquire heme from methemoglobin (metHb) but directly transfers its heme to HtsA. It has not been previously determined whether Shr directly relays heme from metHb to Shp. Thus, the complete pathway for heme acquisition from metHb by the GAS heme acquisition machinery has remained unclear. In this study, the metHb-to-Shr and Shr-to-Shp heme transfer reactions were characterized by spectroscopy, kinetics and protein-protein interaction analyses. Heme is efficiently transferred from the β and α subunits of metHb to Shr with rates that are 7 and 60 times greater than those of the passive heme release from metHb, indicating that Shr directly acquires heme from metHb. The rapid heme transfer from Shr to Shp involves an initial heme donor/acceptor complex and a spectrally and kinetically detectable transfer intermediate, implying that heme is directly channeled from Shr to Shp. The present results show that Shr speeds up heme transfer from metHb to Shp, whereas Shp speeds up heme transfer from Shr to HtsA. Furthermore, the findings demonstrate that Shr can interact with metHb and Shp but not HtsA. Taken together with our published results on the Shp/HtsA reaction, these findings establish a model of the heme acquisition pathway in GAS in which Shr directly extracts heme from metHb and Shp relays it from Shr to HtsA.

## Introduction

Iron is an essential nutrient for growth and survival of most bacterial pathogens. Due to the extremely low solubility of ferric iron under physiological conditions, there is insufficient free iron in hosts to support bacterial growth. The sources of iron *in vivo* for bacteria are host hemoproteins, such as hemoglobin (Hb), haptoglobin, and hemopexin, non-heme iron-protein complex transferrin, and other iron complexes [1]. Heme is a major source of iron for bacterial pathogens. Some bacteria produce hemophore to sequester heme from host hemoproteins [2], [3]. Heme can be directly sequestered from host proteins by receptors on the bacterial surface [4], [5], [6]. Captured heme is transported across the outer membrane by a TonB-dependent process in Gram-negative bacteria [7] or is relayed through the cell wall by surface proteins in Gram-positive pathogens [8], [9]. ATP-binding cassette (ABC) transporters then transport heme across the cytoplasmic membrane.

Group A *Streptococcus* (GAS) is a Gram-positive human pathogen causing a variety of diseases including pharyngitis, cellulitis, necrotizing fasciitis, and streptococcal toxic shock syndrome. GAS uses heme and hemoproteins as sources of the essential iron [10]. The Shr/Shp/HtsABC locus is known to be involved in uptake of heme as an iron source [11], and it encodes the surface proteins Shr and Shp and the ATP-binding cassette transporter HtsABC (also known as SiaABC). Several structural and functional features of these proteins have been established: Shr has two NEAT domains [12]; Shr, Shp, and HtsA, the lipoprotein component of HtsABC, all bind heme [6], [13], [14], [15]; Shp can directly transfer its heme to HtsA [16], [17]; and Shr donates its heme to Shp [6]. However, it is not known whether Shr directly acquires heme from methemoglobin (metHb) and directly transfers it to Shp, and, thus, the pathway of heme acquisition from metHb by the Shr/Shp/HtsABC system has not been established. In this report, we present evidence that supports a model of the Shr/Shp/HtsABC heme acquisition pathway in which Shr directly extracts heme from metHb and delivers it to Shp. Shp relays the heme directly to HtsA.

## Results

### Heme transfer from metHb to apoShr

**Figure 1:**
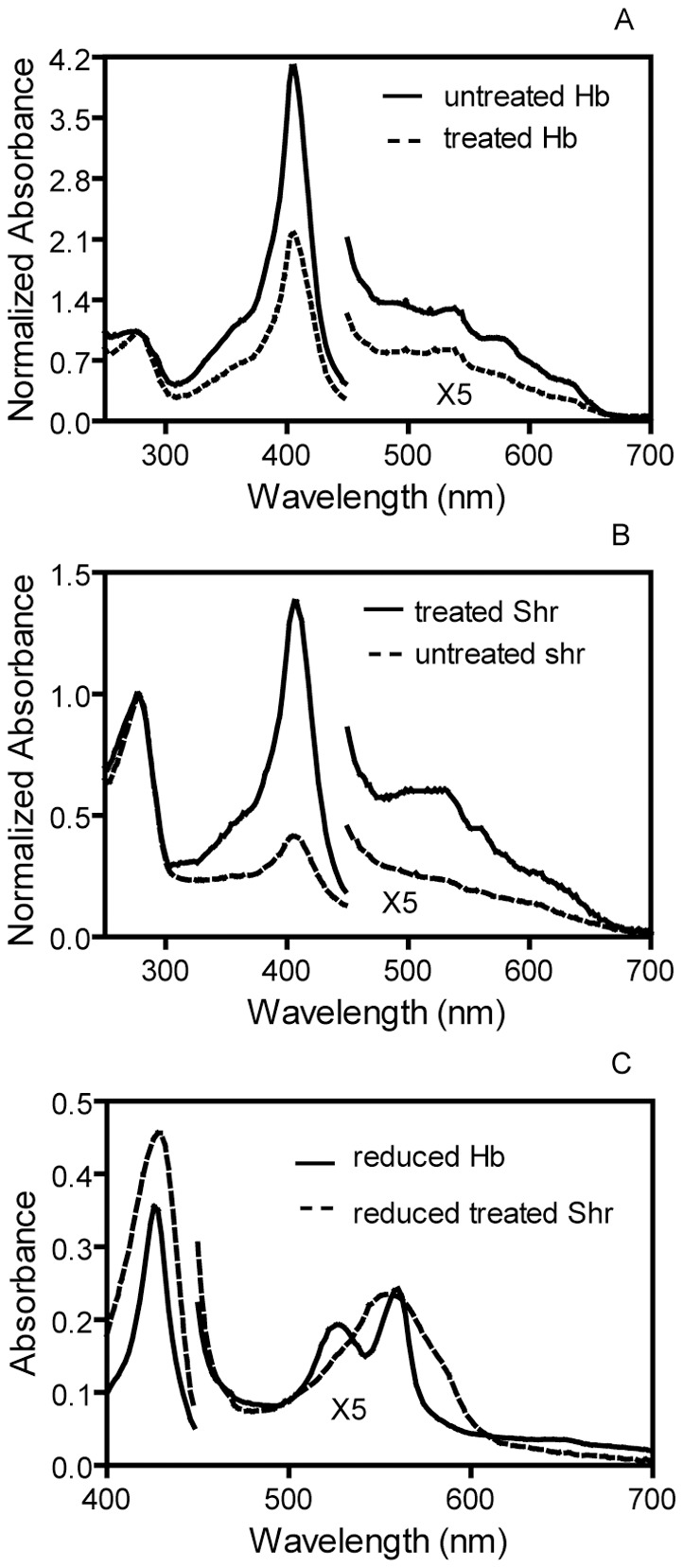
Spectral evidence showing the loss of heme by metHb and the gain of heme by Shr in the metHb/apoShr reaction. (**A**) Absorption spectrum of Hb before and after the reaction of metHb with apoShr. (**B**) Absorption of Shr before and after the reaction of metHb with apoShr. (**C**) Absorption spectrum of reduced Shr isolated from the reaction of metHb with apoShr, and the spectrum of reduced Hb is included for comparison. The spectra in panels A and B were normalized by dividing the absorbance data by the A_280_ value. The spectra of reduced Shr and Hb were recorded in the presence of excess dithionite.

We previously developed a method to demonstrate whether a hemoprotein directly transfers its heme to another protein. The first step in this approach is to demonstrate heme transfer from donor to acceptor by separating the two proteins after reaction and then assessing the loss and gain of heme by the donor and acceptor, respectively, based on the optical absorption spectra of the proteins before and after reaction. The second step is to compare the rate of the heme transfer reaction with that of passive heme release from the heme donor. If the rate of the heme transfer is much faster than that of the passive heme release, heme is directly transferred from the donor to acceptor. This approach was used to determine whether metHb transfers heme to Shr and, if it does, whether the transfer is direct. MetHb (10 µM heme) was incubated with 5.2 µM apoShr in 2 ml Tris-HCl pH 8.0 for 30 min, and the two proteins were separated using SP Sepharose chromatography as described in the Materials and Methods. MetHb does not bind to SP Sepharose but Shr does, allowing efficient separation of the two proteins, which was confirmed by SDS-PAGE (data not shown). The A_406_/A_280_ ratio of metHb after the treatment decreased by 47% (Figure 1A). A_406_ is the Soret absorption of bound heme, and A_280_ is primarily the absorbance of the protein moiety. The decrease in the A_406_/A_280_ ratio of the treated metHb indicates that metHb lost heme in its reaction with apoShr. Consistent with this result, the A_406_/A_280_ ratio of Shr increased from 0.41 before the reaction to 1.38 after the reaction (Figure 1B). This result indicates that apoShr acquired heme in the reaction. The determination of heme content using the pyridine hemochrome assay found that the recovered Hb and Shr samples had 10.2 and 15.4 nmole heme, respectively. The initial Shr sample had 5.2 nmole heme. These measurements indicate that metHb lost 9.8 nmole heme, whereas Shr gained 10.2 nmole heme. Thus, it is likely that apoShr acquired heme from metHb in the reaction.

To further confirm the metHb-to-apoShr heme transfer, the Shr protein recovered from the reaction and metHb were reduced with excess dithionite, and their absorption spectra were recorded. As shown in Figure 1C, the recovered Shr sample shows the well resolved α and β bands at 528 nm and 560 nm. This spectral pattern for hexacoordinate, low spin ferrous heme iron is same as that of reduced holoShr [6] but is different from the spectrum of the pentacoordinate, high spin heme iron of reduced Hb. This result provides solid cross validation that the hemoprotein in the recovered Shr is holoShr and supports the conclusion that apoShr acquired heme from metHb.

### Kinetic evidence for direct heme transfer from metHb to apoShr

**Figure 2:**
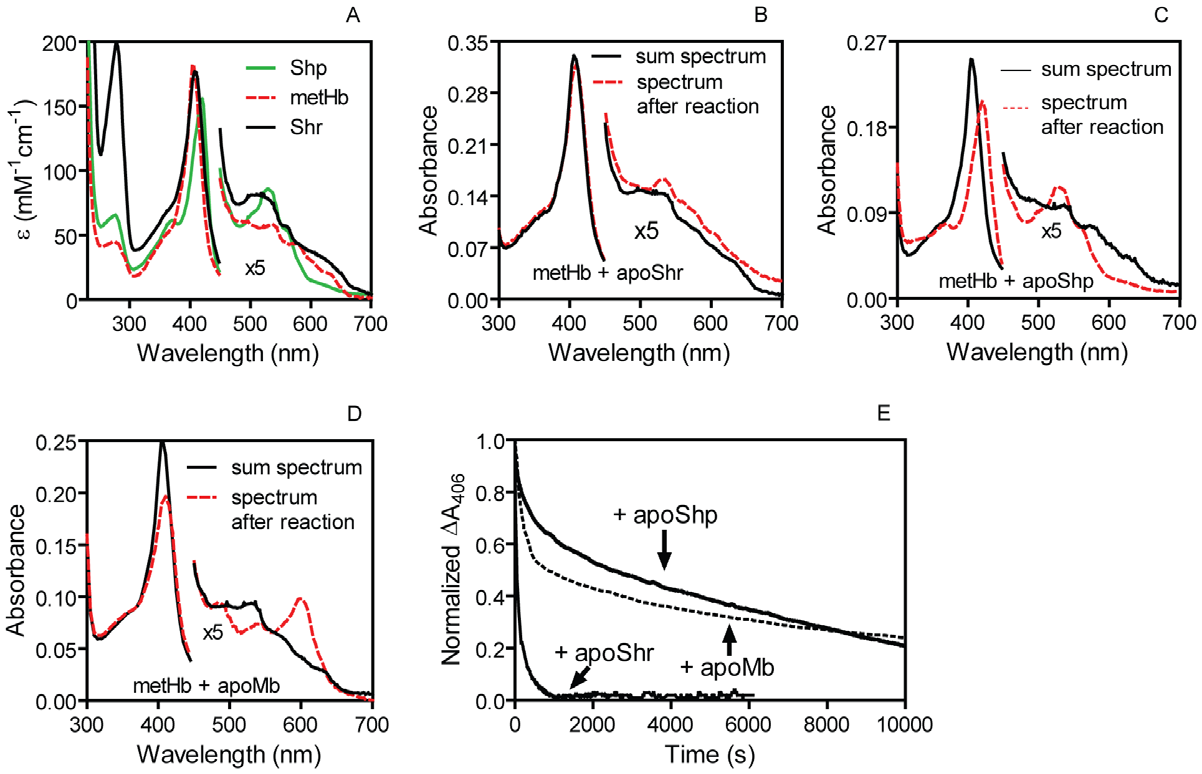
Spectral shift and ΔA_406_ time course in the reaction of metHb with apoShr, apoShp, and H64Y/V68F apoMb. (**A**) Comparison of the spectrum of holoShr, holoShp, and metHb. (**B–D**) The sum of the spectrum of the two individual proteins for and the spectrum of the indicated reaction solution. The higher Soret peak in panel B than panels C and D was due to the presence of holoShr in the apoShr preparation. (**E**) The time course of normalized ΔA_406_ in the three heme transfer reactions. The normalization was done by dividing the observed ΔA_406_ by the maximum ΔA_406_, which were 0.019, 0.12, and 0.065 for the reactions of metHb with apoShr, apoShp, and apoMb, respectively.

Next, we characterized the kinetic parameters of the metHb/apoShr, metHb/apoShp, and metHb/apoMb reactions. Compared with metHb, ferric holoShr and holoShp show 2-nm and 14-nm red shifts in the Soret peak and have a difference in the extinction coefficient at the Soret peak (Δε_Soret peak_) of −5.6 and −26.8 and a Δε_406_ of −8.1 and −67, respectively (Figure 2A). In comparison with the sum absorption spectrum of the two individual proteins in each reaction, metHb/apoShr (Figure 2B), metHb/apoShp (Figure 2C), and metHb/H64Y/V68F apoMb (Figure 2D) reaction solutions after 16-h incubation display the spectral changes that are anticipated for heme transfer from metHb to the heme acceptors. Thus, the time course of ΔA_406_ in these heme transfer reactions can be monitored to compare the kinetics for the reactions of 1.5 µM metHb with 5.2 µM apoShr, 25 µM apoShp, and 50 µM H64Y/V68F apoMb (Figure 2E). The time courses of ΔA_406_ in these reactions fit a double exponential equation and produced two observed rate constants. The metHb/apoShr reaction had rate constants of 0.027 and 0.0042 s^−1^, the rate constants of the metHb/apoShp reaction were 0.0018 and 0.00004 s^−1^, and the metHb/apoMb reaction showed rate constants of 0.0037 and 0.00007 s^−1^. The fast phase of the reaction is apparently for heme transfer from the β subunit of metHb, whereas the slow reaction phase likely reflects heme transfer from the metHb α subunit [18]. Although the apoShr concentration in the reaction was lower than [apoShp] or [apoMb], the observed rates for Shr to obtain heme from the β and α subunits of metHb were 7- and 60-fold higher than those in the metHb/apoMb reaction, respectively. The observed rates in the metHb/apoShp reaction were slow in a manner similar to those in the metHb/apoMb reaction. It is known that apoMb is the scavenger of passively released heme from metHb [18]. Thus, these results indicate that Shr directly acquires heme from metHb.

### Spectral shift and equilibrium of the holoShr-to-apoShp transfer reaction

**Figure 3:**
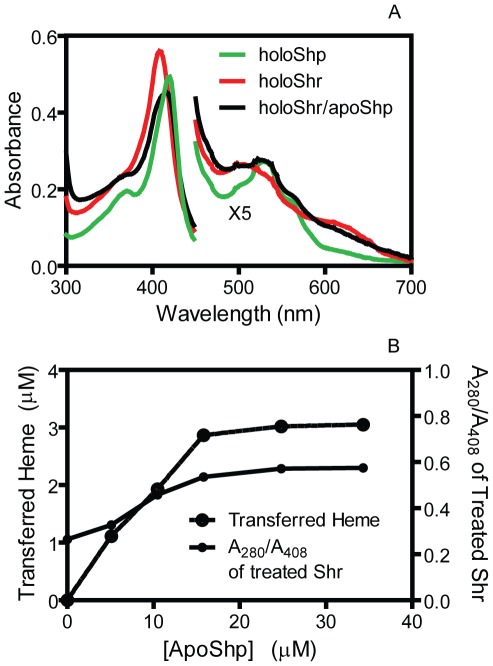
Spectral shift and apoShp titration in heme transfer from holoShr to apoShp. (**A**) Comparison of the absorption spectrum of 3.2 µM holoShr alone with the spectrum of a mixture of 3.2 µM holoShr and 23 µM apoShp. The spectrum of the mixture was taken immediately after mixing, and the spectrum of 3.2 µM holoShp was included for comparison. (**B**) ApoShp titration in heme transfer from holoShr to apoShp. Shown are the A_280_/A_408_ ratio of the recovered Shr and the amount of transferred heme.

We used titration assays to determine the equilibrium constant of the downstream holoShr-to-apoShp heme transfer reaction. The Soret absorption of holoShr and holoShp peaked at 408 nm and 420 nm, respectively (Figure 2A). The absorption spectrum of 3.2 µM holoShr shifted toward the spectrum of holoShp immediately after the addition of small volume of concentrated apoShp, but the resulting spectrum did not perfectly overlap with the spectrum of 3.2 µM holoShp (Figure 3A), indicating that not all holoShr transferred its heme to apoShp. To find the basis for the incomplete heme transfer, we performed apoShp titration for the Shr-to-Shp heme transfer. HoloShr (5.5 µM) was incubated with 5.1, 10.5, 15.8, 24.8, or 34.3 µM apoShp at room temperature for 30 min, and the two proteins were separated. The A_280_/A_408_ ratio of the isolated Shr protein and transferred heme were measured. As shown in Figure 3B, both transferred heme and A_280_/A_408_ increased first with increase in [apoShp] and then reached a plateau when [apoShp] was >15 µM. Approximately 37% of Shr could not transfer its heme to apoShp, which was the apparent reason why the spectrum of the Shr/apoShp reaction mixture did not overlap with the spectrum of holoShp in Figure 3A. The heme-binding domain of Shr is located in the region of amino acids 976–1129, which does not contain a Cys residue. The percentage of non-transferrable holoShr varied in different preparations. Thus, the non-transferrable portion of the Shr heme most likely is crosslinked to the protein during purification, as opposed to a non-transferable Cys-heme form. The concentrations of apo- and holo-forms of the recovered Shr and Shp proteins from the reactions were calculated using the corresponding extinction coefficients and were used to determine the concentrations of apo- and holo-forms of each protein in the reaction at equilibrium. Based on the data, the mean value ± SD of the equilibrium constant for the holoShr-to-apoShp heme transfer reaction was 0.7±0.4.

### Kinetic mechanism of heme transfer from holoShr to apoShp

**Figure 4:**
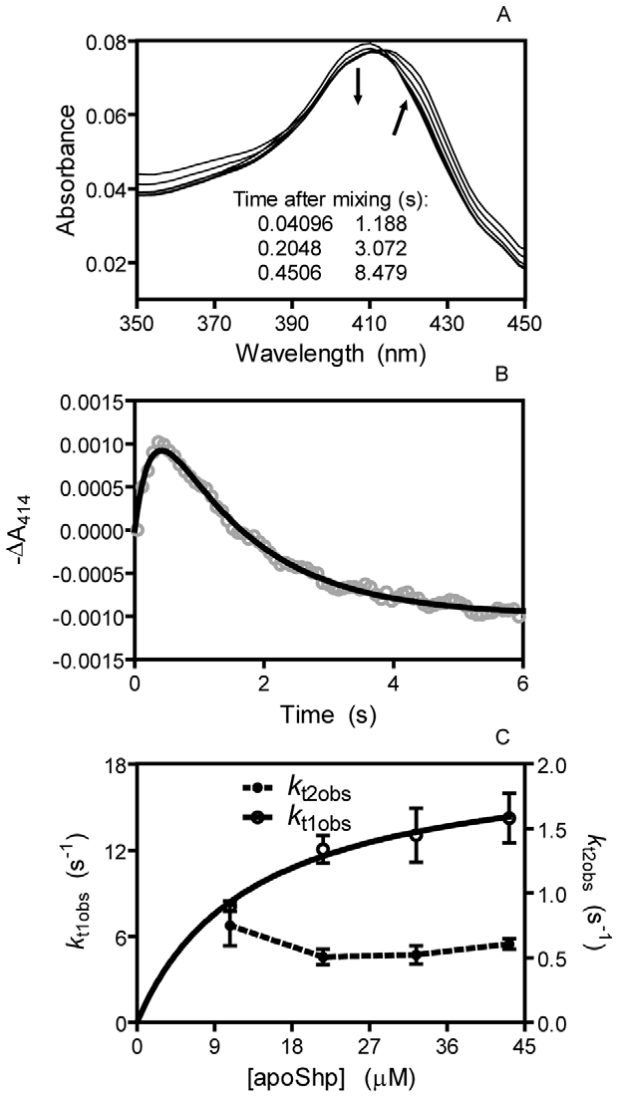
Kinetic mechanism of the holoShr-to-apoShp heme transfer reaction. (**A**) Spectral shift in heme transfer from holoShr to apoShp. Absorption spectra of 0.8 µM holoShr are presented as a function of time for its reaction with 10 µM apoShp. The arrows indicate the directions of the spectral shift with time. (**B**) Time course of −ΔA_414_ in the holoShr/apoShp reaction of panel A. The symbols and line are the observed data and theoretical line obtained by fitting the data to a two-exponential equation. (**C**) The observed rate constants *k*_t1obs_ and *k*_t2obs_ plotted as a function of [apoShp] in the holoShr-apoShp reaction. The rate constants at different [apoShp] were obtained from double-exponential fitting as in B.

To determine whether the Shr-to-Shp heme transfer is direct, we characterized the kinetic mechanism of the holoShr-to-apoShp heme transfer reaction. After mixing holoShr with excess apoShp, the spectrum of the mixture shifted from the spectrum of holoShr to that of holoShp (Figure 4A). Unexpectedly, the absorbance at around 414 nm first decreased and then increased (Figure 4B), and the spectral changes at the other wavelengths of the Soret peaks were also kinetically biphasic. Time courses of ΔA_414_ under pseudo-first order conditions fit a double exponential equation, yielding two observed rate constants (Figure 4B). The observed rate constant at the initial fast phase (*k*_t1obs_) hyperbolically depends on [apoShp], whereas *k*_t2obs_ is independent of [apoShp] and represents a simple first order process (Figure 4C). The initial phase involves the heme donor and acceptor; however, the species formed in this phase of the reaction is an intermediate. The second phase of the reaction represents the formation of the final product but is independent of the apoShp concentration, indicating that the product is converted from a species that already includes the Shp molecule. Taken together, these results suggest that the holoShr-to-apoShp heme transfer proceeds via the species that is formed in the fast phase of the reaction. This interpretation of the kinetic data can be described in terms of the the following minimal model: HoloShr and apoShp first form a complex, and heme transfer begins with the formation of an intermediate, which then converts into apoShr and holoShp in a simple first order process (Scheme S1). When the initial [apoShp] is ≥5[holoShr], the time course for ΔA_414_ can be represented by Equation 1,
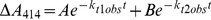
(1)where t is time, and *k*_t1obs_ is given by Equation 2.
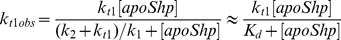
(2)
*K*_d_ equals *k*_2_/*k*_1_, the dissociation constant of the holoShr-apoShp complex. According to this model, *k*_t2obs_ is directly equal to the rate constant *k*_t2_ for the final transfer step to form holoShp. The mean values+SD of *K*_d_, *k*_t1_, and *k*_t2_ were calculated from the data in Figure 3C and are 13.3±4.5 µM, 18.7±3.8 s^−1^, and 0.60±0.14 s^−1^, respectively.

It should be noted that the Scheme S1 does not mean that the two transfer steps are irreversible. The scheme only reflects the interpretation that the rate of heme release from Shp must be small for Eq. 2 to be true and for explaining the independence of *k*_t2_ on [apoShp]. It is also possible that the slow step is not heme transfer within the Shr-heme-Shp complex, but rather a slow heme rearrangement on Shp after heme has been received from Shr.

### Biphasic kinetics of holoHtsA/apoShp reaction

**Figure 5:**
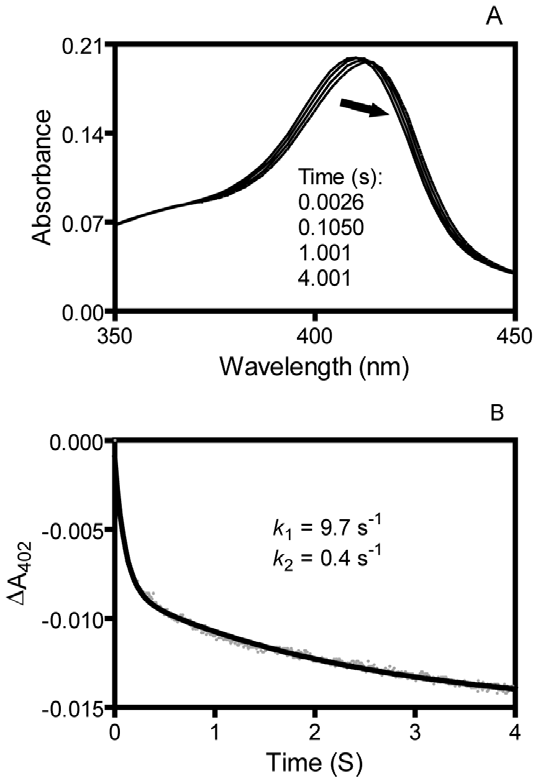
Spectral shift and kinetics of heme transfer from holoHtsA to apoShp. (**A**) Spectral shift in heme transfer from holoHtsA to apoShp. Absorption spectra of 1.3 µM holoHtsA are presented as a function of time for its reaction with 20 µM apoShp. The arrows indicate the directions of the spectral shift with time. (**B**) Time course of ΔA_402_ of the reaction in panel A. The grey and black lines are the observed data and theoretical line obtained by fitting the data to a two-exponential equation, respectively.

To further confirm that the biphasic kinetics is a mechanism for apoShp to acquire heme in a direct heme transfer reaction, we examined the holoHtsA-to-apoShp heme transfer reaction, the reverse reaction of the downstream holoShp-to-apoHtsA reaction. In the downstream reaction, apoHtsA rapidly and directly acquires heme holoShp in a single kinetic phase [17]. The spectrum of holoHtsA in a mixture with excess apoShp rapidly shifted toward that of holoShp (Figure 5A). However, in contrast to the downstream apoHtsA/holoShp reaction, the spectral change in the holoHtsA/apoShp reaction displays two kinetic phases with two observed rate constants of 9.7 s^−1^ and 0.4 s^−1^ in a reaction of 1.3 µM holoHtsA with 20 µM apoShp (Figure 5B). The rate constant of heme dissociation from holoHtsA is 0.0026 s^−1^ as measured using H64Y/V68F apoMb as a heme scavenger [17]. The higher rates of the holoHtsA/apoShp reaction compared with the holoHtsA/apoMb reaction indicate that apoShp directly acquires heme from holoHtsA. Thus, the reactions of apoShp with both holoShr and holoHtsA follow a deliberate biphasic kinetic mechanism.

### Relay role of Shr and Shp in the heme acquisition pathway

**Figure 6:**
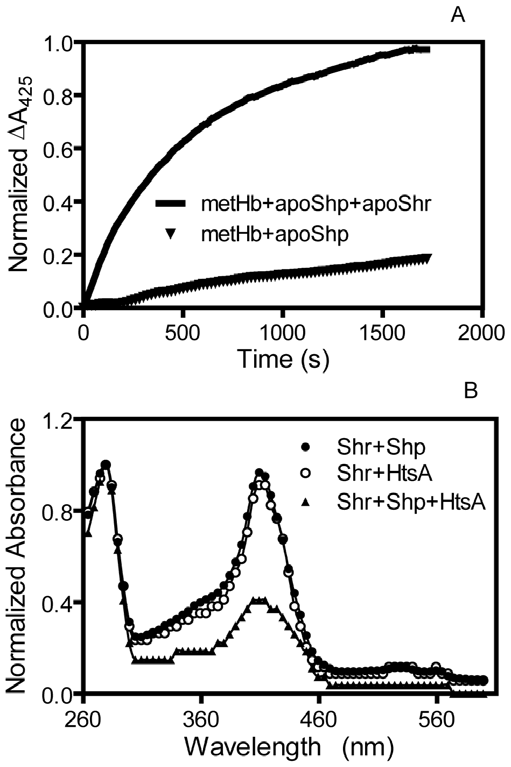
Relay role of Shr and Shp in heme transfer. (**A**) Shr enhances the rate of heme transfer from metHb to apoShp. (**B**) Enhancement of heme transfer from Shr to apoHtsA by Shp.

Heme transfers from metHb to apoShp and from Shr to apoHtsA are both slow and passive [6]. Because Shr and Shp acquire heme more rapidly from their upstream donor and donate it to their downstream acceptors, inclusion of Shr and Shp should enhance heme transfer efficiency. To test the role of Shr as a relay between metHb and Shp, 2.4 µM metHb was reacted with 35 µM Shp with and without 2.0 µM apoShr. ΔA_425_, which represents the formation of holoShp, was recorded over time. The apparent rate of holoShp formation in the presence of apoShr was 20 fold higher than in the absence of apoShr (Figure 6A), demonstrating that Shr speeds up the heme transfer from metHb to Shp.

To determine the relay role of Shp in the holoShr/apoHtsA reaction, 6 µM holoShr was incubated with 18 µM apoHtsA with and without 0.2 µM apoShp for 2 min, and Shr was isolated from the reaction mixture. The absorption spectra of the isolated Shr indicate that majority of holoShr lost its heme in the presence of trace Shp but not in the absence of Shp (Figure 4B). Thus, Shp also speeds up heme transfer from Shr to HtsA.

### Specific interactions among the proteins in the GAS heme acquisition pathway

**Figure 7:**
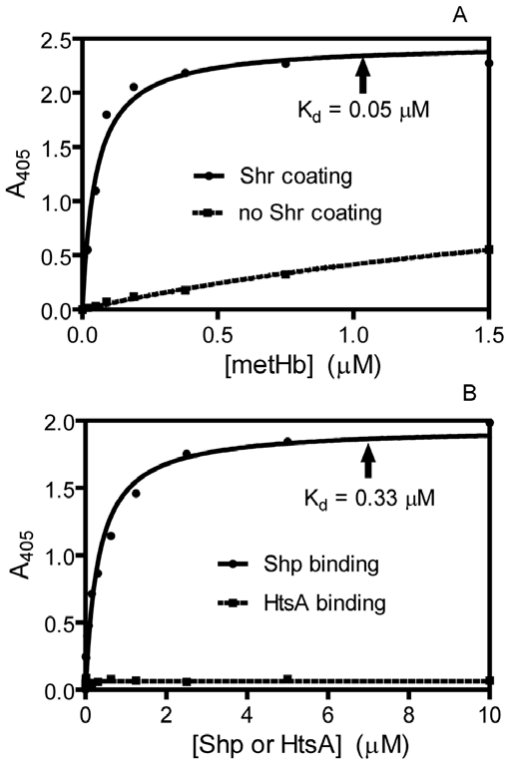
Interaction of Shr with metHb and Shp but not with HtsA. (**A**) ELISA results showing interaction of Shr with metHb. (**B**) ELISA results showing the binding of Shp but not HtsA to immobilized Shr.

The proteins involved in direct heme transfer reactions must interact with each other. An enzyme-linked immunosorbent assay (ELISA) was used to assess interactions among the components of the metHb/Shp/Shp/HtsABC pathway. Shr can form a complex with metHb and Shp with a dissociation constant of 0.05 µM and 0.33 µM, respectively; however, there was no interaction between Shr and HtsA (Figure 7). We have also demonstrated interactions between the Shp and HtsA proteins during heme transfer between holoShp and apoHtsA [14]. These interaction data are consistent with the specific heme transfer results.

## Discussion

This study reports four findings regarding the pathway for heme acquisition from metHb by the Shr/Shp/HtsABC system. First, full-length Shr directly and efficiently acquires heme from metHb. Second, heme is directly transferred from Shr to Shp in a novel biphasic kinetic mechanism. The third finding has two parts: Shr can relay heme from metHb to Shp, and Shp can relay heme from Shr to HtsA. Fourth, the protein-protein interactions among the components of the system are consistent with the direct heme transfer reactions. These findings establish the pathway of the heme acquisition by the GAS heme uptake machinery.

It has been shown by Ouattara et al. that a Shr fragment containing the N-terminal and NEAT1 domains binds metHb and is sufficient to acquire heme from metHb [19]. Based on the difference in the formation of holoNEAT1 after contact with metHb and hemin, Ouattara et al. proposed that this Shr fragment directly acquires heme from metHb. Whether this fragment directly acquires heme from metHb has not been convincingly demonstrated. In addition, the reaction using a truncated protein may not correctly reflect the reaction using the full-length protein. Thus, it is a significant advancement to have found that the full-length Shr protein directly and efficiently acquires heme from metHb in this report.

Shr shows a high affinity for hemoglobin with a K_D_ of 50 nM, which is similar to the affinity of the *Staphylococcus aureus* hemoglobin receptor IsdB for hemoglobin (K_D_ = 55 nM) [20] and the NEAT1 domain of the anthrax hemophore IsdX2 for hemoglobin (K_D_ = 41 nM) [21]. The similar high affinity of these hemoglobin receptor or hemophore for hemoglobin is apparently evolved for capturing hemoglobin. In contrast, Shr has a lower affinity for Shp (K_D_ = 0.33 µM), which could be optimized for the turnover of the heme acquisition reaction.

The detailed kinetic mechanism of direct heme transfer has been described for the Shp/apoHtsA and IsdA/apoIsdC reactions [17], [22]. Both involve a concerted two-step process in which the donor first forms a complex with the acceptor and then donates the heme in a single kinetic phase. The Shr/apoShp reaction displays a different kinetic mechanism that has not been described previously in bacterial heme uptake systems, although a similar mechanism was reported by us for the reactions of the Shp axial ligand deletion mutants with apoHtsA [23]. In this mechanism, Shr and apoShp appear to first form a complex, and the subsequent heme transfer is kinetically biphasic and has a transfer intermediate.

The kinetic mechanism of the reactions of apoShp with holoShr and holoHtsA suggests that apoShp may use one axial ligand to make initial attachment to the heme iron in the donors, then proceeding to the sequential formation of the two axial bonds of the holoShp product. However, alternative explanations should be explored. First, the data could be explained by a mechanism in which the secondary slow step is not heme transfer within the donor-heme-apoShp complex but rather a slow heme rearrangement on Shp after heme has been transferred to it. The spectral change associated with the slow phase of the holoHtsA-apoShp reaction appears to be too big to be due to the rearrangement but to involve replacement of axial ligands instead. Furthermore, the binding of hemin to apoShp is a single exponential process and does not involve rearrangement [17]. Second, the slow step could be due to an indirect heme transfer. However, the slow step is too fast (0.4–0.5 s^−1^) to be due to indirect transfer, given that the rate of the passive heme release from holoShr and holoHtsA is 0.0036 s^−1^ and 0.0026 s^−1^, respectively. Additional experiments are needed to determine the exact basis for the biphasic spectral change.

In contrast, the kinetic studies on the holoShp/apoHtsA reaction [17] support a different mechanism in which the two axial residues in apoHtsA slide along the two sides of the bound heme in Shp and displace the two axial residues of the heme in Shp at about same time. Kinetic studies on the reactions of wild-type and axial mutant Shp proteins with HtsA axial mutants support this sliding mechanism, and docking analysis on Shp and HtsA shows that this mechanism can occur (unpublished results). The kinetic mechanism of the holoIsdA/apoIsdC reaction indicates that the two proteins first form a complex prior to the heme transfer [22]. Formation of the IsdA/IsdC complex is supported by the detection of a transient IsdA-IsdC complex in which the active sites of the heme donor and acceptor are brought together [24]. Apparently, the mechanism of axial displacement following the formation of the heme donor-acceptor complex is not conserved.

Together with the direct heme transfer from Shp to HtsA [17], the two direct transfer reactions demonstrated in this study present *in vitro* evidence that supports an acquisition pathway of direct metHb→Shr→Shp→HtsABC heme transfer for GAS. This pathway model is further confirmed by the relay roles of Shr and Shp in heme transfer from metHb to Shp and from Shr to HtsA, respectively. The interactions between metHb and Shr, between Shr and Shp, and between Shp and HtsA [16] also support the pathway model.

There are parallel functions of the components in the *S. aureus* Isd and *S. pyogenes* Shr/Shp/HtsABC systems. IsdB and Shr capture metHb and extract heme from it, and Shp and IsdA/IsdC relay heme from Shr to HtsA and from IsdB to IsdE, respectively [4], [6], [8], [17], [22], [25]. However, several points indicate that they are two distinct systems. First, the number of the genes involved is different. The *S. pyogenes* system has two surface protein genes, whereas the Isd system has four surface protein genes. Second, the *S. pyogenes* genes are organized as a single operon, whereas the *isdH*, *isdA*, *isdB*, and *isdCDEF* genes are transcribed separately. Third, the two systems do not crosstalk *in vitro*. *S. pyogenes* Shp can directly and rapidly transfer its heme to the HtsA homologue of *Streptococcus equi* [26] but not to IsdE (unpublished data), which is homologous to HtsA. The *Bacillus anthrax* heme acquisition system represents a unique system in Gram-positive pathogens. Although the anthrax system also has the IsdC protein [27], which, like the *S. aureus* IsdC, relays heme from the upstream heme donor to IsdE, the anthrax system uses the hemophores IsdX1 and IsdX2 to capture heme from metHb and deliver it to IsdC [21], [28]. Despite the differences among the three systems, all the non-ABC transporter proteins in these systems use the NEAT domain(s) to interact with other proteins and bind heme. Therefore, all the systems may use the similar biochemical and biophysical mechanisms to transfer heme from one protein to another along the heme acquisition pathway.

## Materials and Methods

### Materials

Rabbit anti-human hemoglobin antiserum was purchased from Sigma. Affinity-purified anti-Shp and anti-HtsA rabbit antibodies have been described [13], [29]. Goat anti-rabbit IgG-HRP conjugate was purchased from Santa Cruz Biotechnology. All solutions were buffered with 20 mM Tris-HCl pH 8.0.

### Protein purification

Recombinant Shr, apoShp and apoHtsA proteins were prepared, as previously described [6], [17], [23]. Purity of Shr, Shp and HtsA proteins was ∼70%, >95%, and >95%, respectively, based on SDS-PAGE analysis. Human hemoglobin was purified as a complex with CO, as described previously (16). MetHb was prepared by oxidizing CO-hemoglobin with ferricyanide and passing the sample through a G-25 column (1.5×30 cm) to remove excess ferricyanide. H64Y/V68F whale sperm apomyoglobin was prepared, as previously described [18].

Purified Shr was in holo-form. Because apoShr was precipitated during freezing and thawing, it was prepared freshly and used right after the final dialysis. About 2 ml 4 µM holoShr was mixed with 1.5 ml 30 µM apoHtsA and 1 µM apoShp, and the mixture was incubated at room temperature for 20 min and then loaded onto a SP Sepharose (0.3 ml resin). The column was washed with 8 ml Tris-HCl and eluted with 150 mM NaCl. The sample was then dialyzed against Tris-HCl overnight. Usually, 70% of the Shr heme was removed, and the remaining heme was apparently crosslinked to Shr since it could not be extracted by the methyl ethyl ketone method [30].

### Determination of protein concentration and heme content

Protein concentrations were determined using the modified Lowry protein assay kit with BSA as a standard. Heme contents of hemoproteins were determined with the pyridine hemochrome assay [31].

### Heme transfer from metHb to apoShr

MetHb at 10.0 µM was incubated with 5.2 µM apoShr in 0.2 ml Tris-HCl at 22°C for 30 min. The reaction mixture was loaded onto a SP Sepharose column (∼0.2 ml resin). The column was washed with 3 ml Tris-HCl, and eluted with 200 mM NaCl. The flowthrough, wash, and elution solutions were collected as 0.3-ml fractions. Hb was recovered in the flowthrough and wash fractions, and Shr was in elution according to SDS-PAGE results. Absorption spectra of metHb and Shr before and after reaction were recorded to assess heme transfer from metHb to apoShr. The spectra were normalized by dividing the absorbance data by the A_280_ value. The spectra of reduced Shr and Hb were recorded in the presence of excess dithionite.

### Kinetic analysis of heme transfer from metHb to apoShr, apoShp and apoMb

The change in A_406_ was monitored over time after mixing 1.5 µM metHb with 5.2 µM apoShr, 25 µM apoShp and 50 µM apoMb using a Spectra^Max^ spectrophotometer (Molecular Devices). The ΔA_406_ data in the reactions of metHb with apoShr, apoShp, and apoMb were normalized by dividing ΔA_406_ by 0.019, 0.12, and 0.065, respectively. The normalized ΔA_406_ time courses were analyzed by fitting to a double exponential equation using the version 5 GraphPad Prism Software. Rate constants were obtained as described in the Results

### ApoShp titration for the Shr-to-Shp heme transfer reaction

HoloShr (5.5 µM) was incubated with 5.1, 10.5, 15.8, 24.8, and 34.3 µM apoShp in 0.2 ml 20 mM Tris-HCl pH 8.0 at 22°C for 30 min, and the two proteins were separated using a small SP Sepharose column (∼0.1 ml resin) as described previously [6]. The concentrations of holoShr and holoShp were calculated from A_408_ and A_420_ using the extinction coefficient of 1.74×10^5^ and 1.55×10^5^ M^−1^•cm^−1^, and the concentrations of heme-transferrable holoShr were obtained by excluding the non-transfer heme, 37% of initial [holoShr]. The concentrations of apoShr and apoShp in the recovered Shr and Shp samples were calculated from A_280_ after subtracting the contribution from holo-form using the extinction coefficients of 1.88×10^5^ and 5.6×10^4^ M^−1^•cm^−1^.

### Kinetics of heme transfers from Shr and holoHtsA to apoShp

A stopped-flow spectrophotometer equipped with a photodiode array detector (SX20, Applied Photophysics) was used to measure the rates of heme transfer from holoShr and holoHtsA to apoShp. HoloShr (0.8 µM) or holoHtsA (2.6 µM) in one syringe was mixed with apoShp at ≥5[holoShr] or 40 µM apoShp in another syringe. Absorption spectra were recorded over time. Time courses of the absorbance changes were fitted to Eq 1 using GraphPad Prism software to obtain the observed rate constants for the analysis described in the Results.

### Relay role of Shp in heme transfer from Shr to HtsA

To examine the relay role of Shp in heme transfer from Shr to HtsA, two reactions were set up: One with 6 µM holoShr and 18 µM apoHtsA and another with 6 µM holoShr, 18 µM apoHtsA, and 0.2 µM apoShp. The reaction solutions at 2 min after mixing were loaded onto a 0.2-ml SP Sepharose column. The column was washed with 4 ml Tris-HCl and eluted with 1 ml 200 mM NaCl to recover Shr. The absorption spectra of the Shr in the elution were recorded to assess the loss of heme by Shr. Time courses of ΔA_425_, representing the formation of holoShp, were monitored and normalized to ΔA_425_ after 16 h of reaction time.

### Detection of protein-protein interaction using ELISA

Immulon plates were coated with 150 µl of 20 µg holoShr/ml in PBS or PBS (control) at 4°C overnight. The plates were washed three times with PBS containing 0.05% Tween 20 (TPBS) and were blocked with 2% bovine serum albumin (BSA) at room temperature for 1 h. The plates were incubated with 100 µl metHb, Shp, or HtsA at concentrations from 0 to 10 µM at room temperature for 30 min. After washing with TPBS four times, the plates were incubated for 1 h with 100 µl rabbit anti-human hemoglobin antisera, rabbit anti-Shp antibodies (1 mg/ml) or rabbit anti-HtsA antibodies (1 mg/ml) at 1∶3000 dilution in 0.5% BSA, washed with TPBS 4 times, and incubated with 100 µl goat anti-rabbit IgG-HRP conjugate at 1∶4000 dilution at room temperature for 1 h. The plates were then washed with TPBS 4 times and with PBS 3 times. The reactions were developed using ABTS solution containing 0.01% H_2_O_2_. A_405_ was measured after 20 min of incubation.

## Supporting Information

Scheme S1 **A minimal reaction model for the kinetics of the holoShr-to-apoShp heme transfer reaction.** The *k*_1_ and *k*_2_ constants are the rate constants for bimolecular formation and unimolecular dissociation of the initial holoShr-apoShp complex, respectively, and *k*_t1_ and *k*_t2_ are the first order rate constants for the formation of the intermediate and the products, respectively.
(TIF)
