# Supplementary figures and images for: Direct Heme Transfer Reactions in the Group A Streptococcus Heme Acquisition Pathway
Source: PLoS One. 2012 May 23;7(5):e37556. doi: 10.1371/journal.pone.0037556 (PMC3359286; doi:10.1371/journal.pone.0037556)

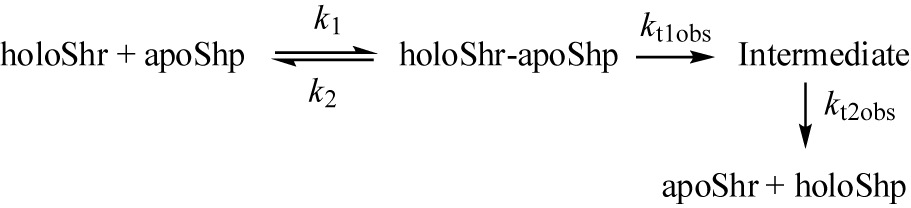

Supplement: Scheme S1 — A minimal reaction model for the kinetics of the holoShr-to-apoShp heme transfer reaction. The k 1 and k 2 constants are the rate constants for bimolecular formation and unimolecular dissociation of the initial holoShr-apoShp complex, respectively, and k t1 and k t2 are the first order rate constants for the formation of the intermediate and the products, respectively. (TIF) [file pone.0037556.s001.tif]
